# Turtle soup, Prohibition, and the population genetic structure of Diamondback Terrapins (*Malaclemys terrapin*)

**DOI:** 10.1371/journal.pone.0181898

**Published:** 2017-08-09

**Authors:** Paul E. Converse, Shawn R. Kuchta, J. Susanne Hauswaldt, Willem M. Roosenburg

**Affiliations:** 1 Department of Biological Sciences, Ohio University, Athens, Ohio, United States of America; 2 Ohio Center for Ecology and Evolutionary Studies, Ohio University, Athens, Ohio, United States of America; 3 Department of Biological Sciences, University of South Carolina, Columbia, South Carolina, United States of America; National Cheng Kung University, TAIWAN

## Abstract

Diamondback terrapins (*Malaclemys terrapin*) were a popular food item in early twentieth century America, and were consumed in soup with sherry. Intense market demand for terrapin meat resulted in population declines, notably along the Atlantic seaboard. Efforts to supply terrapins to markets resulted in translocation events, as individuals were moved about to stock terrapin farms. However, in 1920 the market for turtle soup buckled with the enactment of the eighteenth amendment to the United States’ Constitution—which initiated the prohibition of alcoholic drinks—and many terrapin fisheries dumped their stocks into local waters. We used microsatellite data to show that patterns of genetic diversity along the terrapin’s coastal range are consistent with historical accounts of translocation and cultivation activities. We identified possible instances of human-mediated dispersal by estimating gene flow over historical and contemporary timescales, Bayesian model testing, and bottleneck tests. We recovered six genotypic clusters along the Gulf and Atlantic coasts with varying degrees of admixture, including increased contemporary gene flow from Texas to South Carolina, from North Carolina to Maryland, and from North Carolina to New York. In addition, Bayesian models incorporating translocation events outperformed stepping-stone models. Finally, we were unable to detect population bottlenecks, possibly due to translocation reintroducing genetic diversity into bottlenecked populations. Our data suggest that current patterns of genetic diversity in the terrapin were altered by the demand for turtle soup followed by the enactment of alcohol prohibition. In addition, our study shows that population genetic tools can elucidate metapopulation dynamics in taxa with complex genetic histories impacted by anthropogenic activities.

## Introduction

Turtle soup was a popular food item in the United States during late nineteenth and early twentieth centuries. Although many turtle species were consumed, diamondback terrapins (*Malaclemys terrapin*) were considered a delicacy and were highly sought. The historical market price for terrapins demonstrates their popularity: a dozen larger terrapin sold for $70.00 USD during 1915–1920 [1], or ~$852 in 2017 USD. Recipes for turtle soup varied, but many contained sherry. However, in 1920 the United States ratified the Eighteenth Amendment, banning the production, sale, and transport of alcoholic beverages (Prohibition). The sherry used to make turtle soup became difficult to procure, and demand for turtle soup plummeted. After the market crashed, several terrapin farms purportedly dumped their stocks into local waters.

Prior to Prohibition, intense demand for turtle soup resulted in the decline of terrapins across large portions of their range, particularly along the Atlantic seaboard [2]. Due to their larger size, female terrapins were preferred [1]. To combat extirpation and supplement the turtle soup market, terrapin farms were established by private businesses and governmental agencies to explore domestication and cultivation [3–6] Terrapins from the mid- and north Atlantic were preferred for their superior taste and size. In particular, terrapins from Chesapeake Bay were the favorite and were sold with the moniker “Chesapeakes” [1, 3]. In 1891, approximately 40,000 kg of terrapin were harvested from Chesapeake Bay alone, but by 1920 terrapin harvests plummeted to ~370 kg [7]. As wild stocks dwindled, terrapins were translocated from other regions in an effort to maintain stock solvency [1]. Terrapins from North and South Carolina (“Carolinas”) were frequently shipped to other states, while terrapins from Florida were derided as too small and insipid in flavor [3]. Terrapins from Texas achieved a desirable size but lacked the flavor of “Chesapeakes” and “Carolinas,” and some terrapin farms hybridized terrapins with the goal of creating a quick-growing, yet flavorful, terrapin [6]. Hybridization experiments between terrapins from Texas and the Carolinas began in 1914 at the U.S. Fisheries Biological Station in Beaufort, North Carolina, and the first hybrid individuals were confirmed in 1919 [6]. However, Prohibition followed these hybridization experiments. As terrapin farms along the Atlantic coast closed, they purportedly released their mixed stocks into local waters. The population admixture that resulted is poorly known.

Previous work has detected hints of the influence of translocation on patterns of terrapin genetic diversity [8]. Terrapins from South Carolina were shown to be more similar to Texas populations, while Florida populations were identified as genetically distinct [9–11]. In addition, dramatic increases in contemporary gene flow into Chesapeake Bay were interpreted to be the result of translocation [12]. Alternatively, documented patterns of genetic diversity could be due to natural movements. For example, the Suwannee Seaway is a hypothetical embayment that existed during the early Miocene and late Pliocene [13–16]. This seaway ran through the northern part of present-day Florida, potentially facilitating gene flow between the Gulf and the Atlantic while isolating populations in peninsular Florida [8].

Here we use microsatellite data to examine patterns of genetic variation across the terrapins’ range and report evidence of human-mediated gene flow that is consistent with historical accounts of terrapin translocation. We accomplish this by: i) inferring population structure in a Bayesian framework, as well as with discriminate analysis of principal components (DAPC); ii) estimating both historical and contemporary levels of gene flow and comparing them to infer changes in gene flow over time; iii) testing alternative models of population structure, including stepping-stone models and models that include translocation events; and iv) surveying for population bottlenecks. Based on previous studies and historical documentation, we hypothesized: i) admixture between Atlantic and Gulf populations, but not with Florida populations; ii) some non-adjacent populations (e.g. Texas and New Jersey) would show increases in contemporary gene flow, consistent with translocation; iii) population genetic models incorporating translocation would outperform other models; and iv) genetic tests for bottlenecks would fail to detect population contractions in populations known to have experienced serious decline, due to human-mediated influxes of genetic diversity.

## Methods

### Sampling and population structure

**Fig 1:**
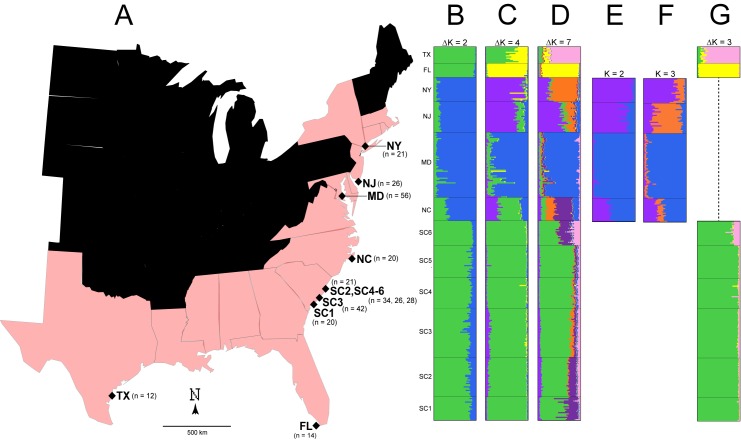
(A) The Gulf and Atlantic seaboards of the Eastern United States. States harboring terrapin populations along their coastlines are faded red. (B) All terrapin populations under ΔK = 2. (C) All terrapin populations under ΔK = 4. (D) All terrapin populations under ΔK = 7. Subsections B,C, and D depict the same analysis, explored under different genotypic partitions. (E) Mid- and north Atlantic terrapins under K = 2. In this scenario, NY and NJ terrapins constitute a single population. (F) Mid- and north Atlantic terrapins under K = 3. In this scenario, NY and NJ form separate populations. Subsections F and G depict the same analysis under two values of K with similar likelihood scores. (G) SC terrapins investigated with Gulf populations. TX and FL form separate populations and SC1-6 constitute a single cluster. No substructure is present within SC1-6. TX, FL, SC, and MD are diagnosable clusters. If NY and NJ form a single cluster (subsection E), there are five total clusters. If NY and NJ form separate clusters (subsection F), there are six total clusters.

We analyzed six polymorphic microsatellite loci (TerpSH1, TerpSH2, TerpSH3, TerpSH5, TerpSH7, TerpSH8) [17] from 12 sampling localities throughout the Gulf and Atlantic seaboards and sampled a total of 320 individuals. (Fig 1A). Individuals were sampled using blood or leg muscle tissue, and DNA isolated using a phenol-chloroform extraction. This dataset includes sampling from Texas (TX), Florida (FL), South Carolina (SC), North Carolina (NC), Maryland (MD), New Jersey (NJ), and New York (NY) during 2001 (Fig 1A). This dataset was previously analyzed for tests of allelic richness, heterozygosity, and number of alleles and can be found in [9]. All sampling protocols and specimen handling followed Ohio University IACUC number L02-06, protocol 13-L-023.

We demarcated coastal population structure with two methods. The first was a Bayesian analysis in the program STRUCTURE v. 2.3 [18]. The second was a discriminate analysis of principal components (DAPC) in the R package adegenet v. 1.4–2 [19]. STRUCTURE delineates genetic clusters by maximizing conformity to Hardy-Weinberg equilibrium while simultaneously minimizing linkage disequilibrium among loci for K user-defined clusters, while DAPC utilizes k-means to maximize variation between groups after PCA transformation. For STRUCTURE, we ran K from 1 to 12 (sampling locations), replicating each value of K ten times, each with a random starting seed. Individual runs were composed of a Markov chain Monte Carlo (MCMC) algorithm of 700,000 steps, with the first 50% removed as burn-in. We used the admixture model, the LOCprior, and LOCISPOP prior for all runs. Preferred values of ΔK were computed with the Evanno method [20] in STRUCTURE HARVESTER v. 0.6.94 [21]. Output from STRUCTURE HARVESTER was processed in CLUMPP v. 1.1.2 [22] to control for labeling switching and multimodality. DISTRUCT v. 1.0 [23] was used to visualize the data. We ran STRUCTURE in a hierarchical fashion: first we ran an initial analysis to detect basal levels of structure [20], and then we searched for additional structure within each identified population individually.

For DAPC, we first optimized the number of PC axes to avoid under- or over-fitting our population genetic models. We accomplished this with cross-validation, which uses stratified random sampling and divides the dataset into a training set and a validation set. We partitioned the training set to be 50% of the data and the validation set to 50%, and employed 100 replicates. The cross-validation method estimated 60 axes should be retained. The Bayesian information criterion (BIC) was used to choose the optimum number of clusters for DAPC analysis. Four discriminate functions (DF) were retained for each analysis.

### Historical and contemporary gene flow

We used MIGRATE v. 3.6.5 [24] to estimate historical gene flow levels (M = m_h_/μ: proportion of migrants per generation, scaled by mutation rate). For these analyses we treated sampling localities as populations with the exception of South Carolina. Because South Carolina lacked structure among its sampling localities (Fig 1G; see results), we treated all South Carolina sampling localities as a single population. For each population, we randomly subsampled 40 individuals for gene flow estimates. If a population had fewer than 40 individuals, we included all individuals in gene flow estimates. For each run, we slice sampled the posterior distribution with a MCMC of 5,000,000 steps with the first 50% removed as burn-in. Each MCMC consisted of four statically heated parallel chains sampled every 500 iterations. Five independent replicates were run, totaling 25,000,000 MCMC steps. Estimates of θ (= 4N_e_μ: effective population size, scaled by mutation rate) were modeled under a uniform distribution bounded between 0.0001 and 100, and historical gene flow estimates were bounded between 0 and 2000.

For estimates of contemporary gene flow (m: proportion of migrants per generation), we used BAYESASS v. 3.0 [25]. We ran 10 independent MCMC simulations with random starting seeds [26] for 30,000,000 steps, and sampled every 3,000 steps. We discarded the first 50% as burn-in. We used TRACER v. 1.5 [27] to visualize MCMC simulations, and used R scripts to calculate a Bayesian deviancy measure to determine the run that best fit the data [28, 29].

### Temporal changes in gene flow

Because MIGRATE uses the coalescent, it estimates gene flow over long periods of time, approximately 4N_e_ generations (several thousand years) in the past [24]. Thus, its gene flow estimates pre-date translocation events in the early twentieth century. By contrast, BAYESASS estimates gene flow “…over the last several generations” [25], where “several” is commonly interpreted as roughly five generations (e.g. [12, 30] With a generation time of 12 years (Roosenburg, unpublished data), the contemporary gene flow estimates of BAYESASS are within the last 60 years or so. These estimates post-date known terrapin translocation events.

To estimate temporal changes in gene flow, we compared historical and contemporary estimates. First, we multiplied the historical gene flow estimates from MIGRATE (M = m_h_/μ) by a mutation rate (μ) of 2.72 x 10^−3^ mutations site^-1^ generation^-1^. This mutation rate was estimated explicitly for the microsatellites used in previous studies [31]. The resulting values are subtracted from the contemporary gene flow estimates (m) generated by BAYESASS. Thus, Δm = m—m_h_. Positive values of Δm indicate increased levels of contemporary gene flow, negative values indicate reduced contemporary gene flow, and values near zero indicate no temporal change in gene flow.

### Testing coastal population structure

**Fig 2:**
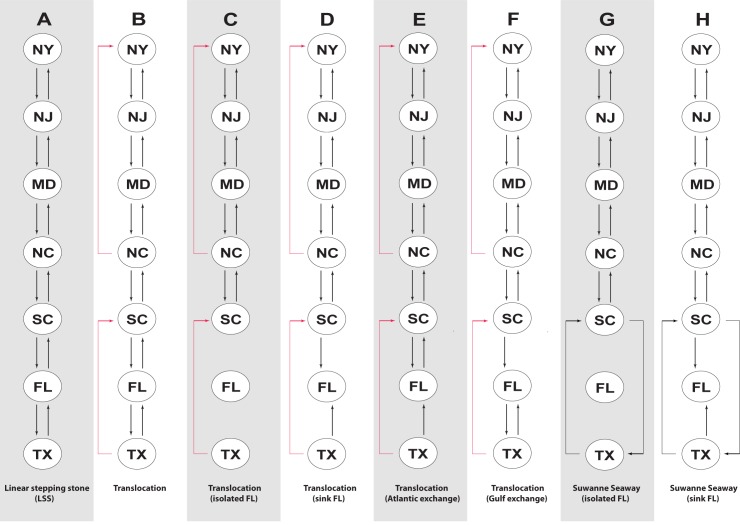
The eight models of population structure derived from STRUCTURE, DAPC, and Δm results. Black lines denote naturally occurring gene flow while red lines indicate gene flow possibly arising from translocation. Model A is a linear stepping-stone; Model B added gene flow from TX to SC and from NC to NY; Model C removed all gene flow to and from FL while retaining translocation; Model D treated FL as a sink population; Model E allowed gene flow with FL on the Atlantic coast; Model F allowed gene flow with FL on the Gulf Coast; Model G depicts the Suwannee Seaway with completely isolated FL populations; Model H depicts the Suwannee Seaway with FL as a sink population.

We compared eight models of gene flow in MIGRATE, generated with the methods described above (Fig 2). Our initial model was a linear stepping-stone (Model A), which restricted gene flow to adjacent populations. We then added or removed gene flow routes from Model A based on results from STRUCTURE, DAPC, and our Δm estimates. Models B–F incorporated gene flow from translocation events with varying connectivity to Florida. Models G and H modeled the Suwannee Seaway. We used approximate Bézier scores and log Bayes factors (LBF) to determine which model best explained coastal population structure.

### Bottlenecks

BOTTLENECK v. 1.2.02 [32] was used to test for bottlenecks. Locus mutation was modeled with the stepwise-mutation model (SMM) and the two-phase model (TPM). The TPM modeled 95% of mutations as single-step while multi-step mutations were modeled with 12% variance [32]. Each test consisted of 20,000 permutations. We tested for bottlenecks by sampling locality and by STRUCTURE cluster. We used the Wilcoxon signed-rank test, which detects bottlenecks 25–250 generations in the past [33], and a mode-shift test, which detects bottlenecks “…within the past few dozen generations” [34].

## Results

### Population structure—STRUCTURE

Initial runs of STRUCTURE found ΔK = 2 best describes coastal population structure (Fig 1B). However, ΔK scores of 4 and 7 are comparable, and we also report them for comparative purposes (Fig 1C and 1D). As ΔK identifies basal levels of hierarchical structure [20], we also tested for substructure within groups for ΔK = 2, which included northern and southern groups. Inference of population substructure within the northern group (NC, MD, NJ, NY) revealed K = 2 or K = 3 subclusters (Fig 1E and 1F). This is due to two solutions of K having overlapping likelihood scores (S1 Fig). If K = 2 is adopted, NJ and NY constitute a cluster (Fig 1E), while if K = 3 is adopted, they form separate subclusters (Fig 1F). The southern group (TX, FL, SC) included ΔK = 3 subclusters (Fig 1G), one for each state. We did not detect substructure among SC sampling localities. Thus, in total there are either five or six genotypic clusters, dependent upon K = 2 or K = 3 for north Atlantic terrapins (S1 Fig).

### Population structure—DAPC

**Fig 3:**
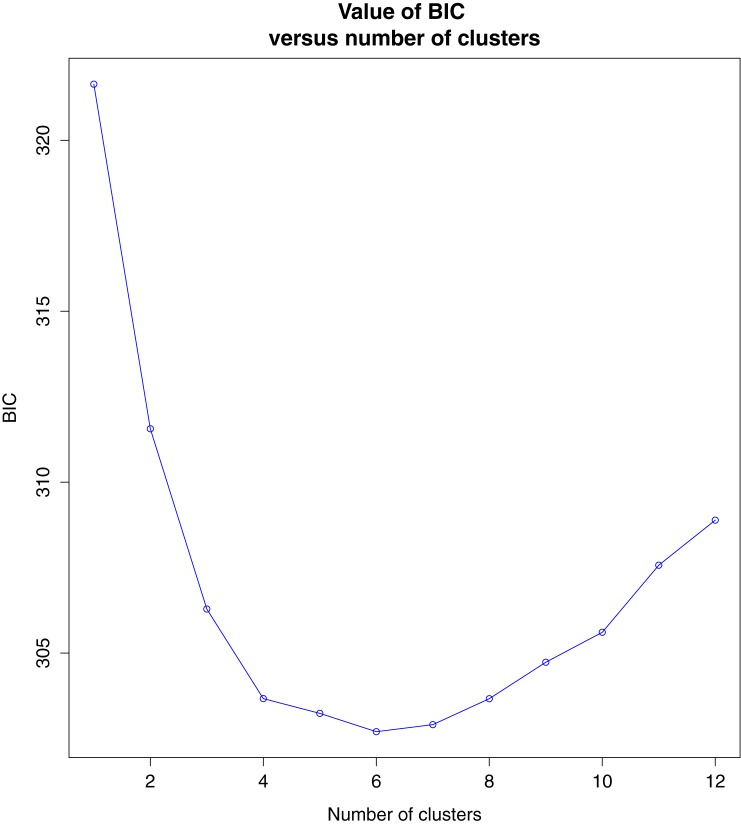
BIC scores indicating k = 6 is the preferred value of genetic clusters when retaining 60 PC axes.

**Fig 4:**
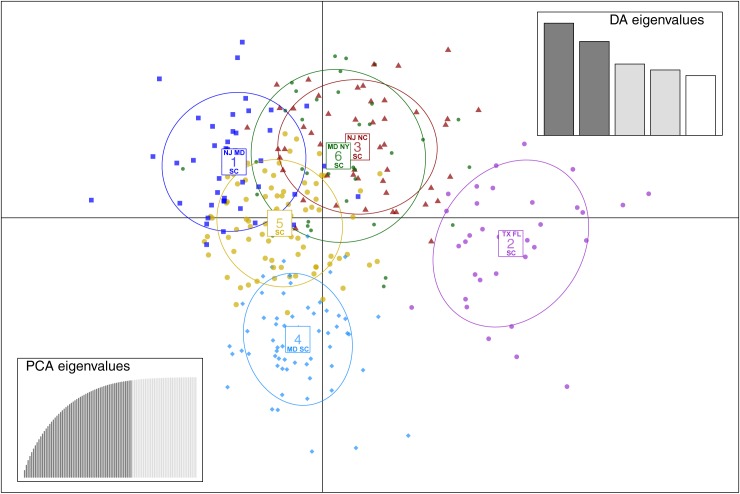
DAPC for 60 retained axes and four discriminate functions. Six clusters are recovered with this model. The top half contains populations along the Atlantic coast while Gulf populations are found on the right half of the diagram. SC terrapins are present within each cluster.

**Fig 5:**
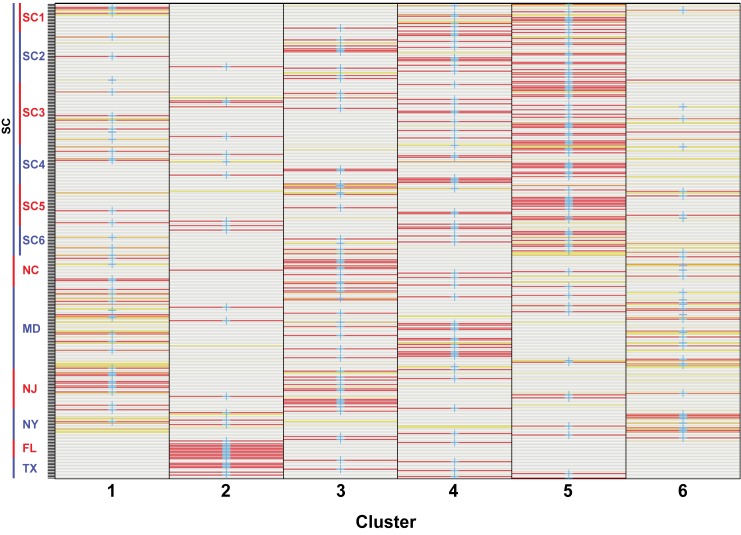
Membership probabilities for 60 retained PC axes and six genetic clusters. Warmer colors denote more certainty in membership probabilities to each respective cluster. Cluster numbers correspond with populations denoted in Fig 4.

BIC indicated the optimum number of clusters is six (Fig 3). DAPC for the six-cluster analysis is shown in Fig 4, and membership probabilities are provided in Fig 5. Mid- and north Atlantic terrapins form clusters 1, 3, and 6; cluster 4 diagnoses Chesapeake Bay (MD) terrapins, and cluster 2 includes populations located in the Gulf (TX, FL). Cluster 5 is composed of SC terrapins and links cluster 4 with clusters 1, 3, and 6. Cluster 2 exhibits no overlap with any cluster.

**Fig 6:**
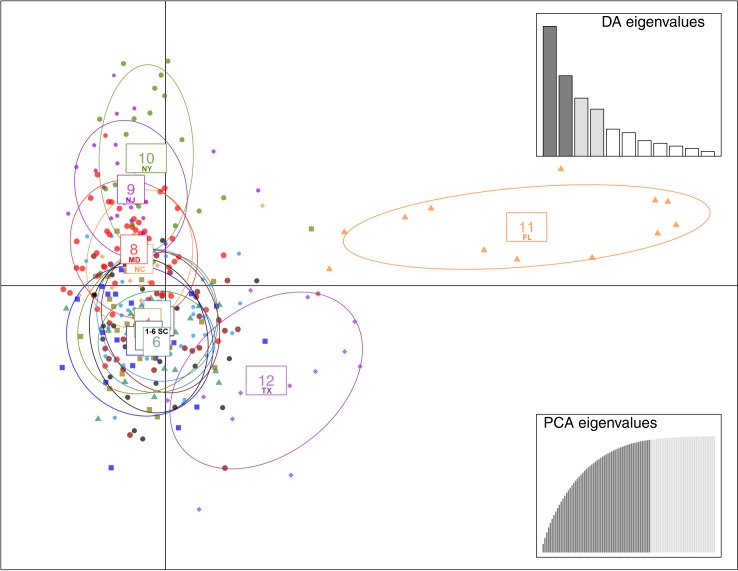
DAPC for 60 retained axes on sampling localities (k = 12). Clusters in the model resemble the spatial distribution of sampling localities along the Gulf and Atlantic seaboards (see Fig 1). FL exhibits no admixture with any cluster while TX shows overlap with populations found in SC.

**Fig 7:**
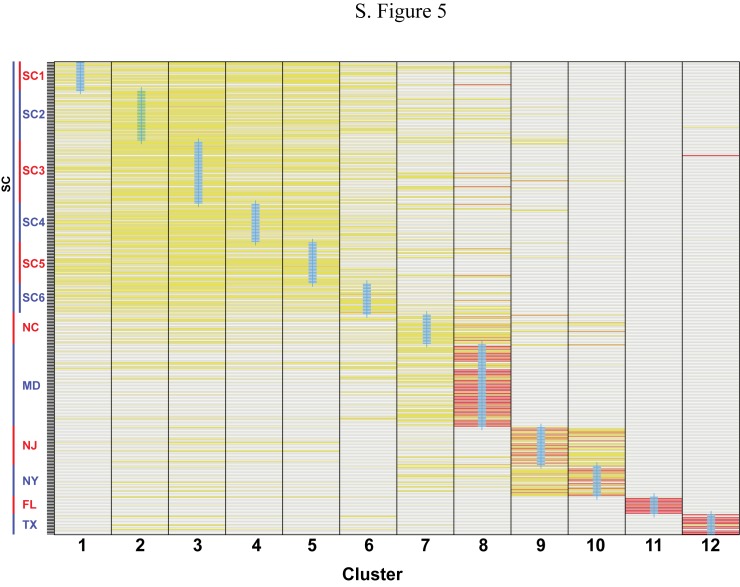
Membership probabilities for 60 retained PC axes and 12 genetic clusters (sampling localities). Warmer colors denote more certainty in membership probabilities to each respective cluster. Cluster numbers correspond with populations denoted in Fig 6.

If DAPC is run on sampling localities (Fig 1A), an alternative population genetic structure is delimited that resembles the spatial distribution of our sampling localities (Figs 6 and 7). Clusters 1–6 (SC1–6) overlap heavily. Cluster 7 (NC) deviates slightly from clusters 1–6. Cluster 8 (MD) overlaps with cluster 7 and clusters 1–6. Cluster 9 (NJ) falls above cluster 8, and cluster 10 (NY) falls above cluster 9. Cluster 11 (FL) exhibits no overlap with any cluster, while cluster 12 (TX) overlaps with SC populations.

### Historical and contemporary gene flow

The highest levels of historical gene flow were from NJ to NC (Table 1; m^h^ = 0.0775) and the lowest were from NY to FL (m^h^ = 0.0128). Contemporary gene flow estimates show SC to NC exhibited the highest levels of gene flow (Table 2; m = 0.1497), while SC to FL demonstrated the lowest levels (m = 0.0037).

**Table 1:** Historical gene flow (m_h_) estimates from MIGRATE among coastal populations; gene flow is measured by the proportion of migrants per generation, ranging from 0.0–1.0. Populations on the left are sending gene flow into recipient populations listed above columns.

Historical gene flow estimates.							
	TX	FL	SC	NC	MD	NJ	NY
TX	-	0.0295	0.0282	0.0235	0.0272	0.0228	0.0520
FL	0.0502	-	0.0224	0.0300	0.0243	0.0232	0.0262
SC	0.0424	0.0263	-	0.0489	0.0287	0.0294	0.0729
NC	0.0354	0.0207	0.0215	-	0.0221	0.0438	0.0254
MD	0.0180	0.0277	0.0719	0.0249	-	0.0451	0.0583
NJ	0.0606	0.0202	0.0421	0.0775	0.0493	-	0.0388
NY	0.0336	0.0128	0.0335	0.0374	0.0280	0.0214	-

**Table 2:** Contemporary gene flow (m) estimates from BAYESASS among coastal populations; gene flow is measured by the proportion of migrants per generation, ranging from 0.0–1.0. Populations on the left are sending gene flow into recipient populations listed above columns.

Contemporary gene flow estimates.							
	TX	FL	SC	NC	MD	NJ	NY
TX	-	0.0268	0.0849	0.0184	0.0191	0.0207	0.0197
FL	0.0207	-	0.0233	0.0181	0.0192	0.0172	0.0195
SC	0.0042	0.0037	-	0.0047	0.0054	0.0077	0.0051
NC	0.0139	0.0133	0.1497	-	0.0511	0.0245	0.0574
MD	0.0169	0.0080	0.0518	0.0101	-	0.0145	0.0129
NJ	0.0108	0.0109	0.0389	0.0154	0.0630	-	0.1125
NY	0.0142	0.0128	0.0324	0.0157	0.0286	0.0303	-

Of the 42 gene flow routes, six exhibited increased levels of contemporary gene flow (Table 3; +Δm > 0.010), 22 demonstrated reduced levels of contemporary gene flow (-Δm < -0.010), and 14 were relatively stable over time (-0.010 < Δm < 0.010). Four of the six gene flow routes estimated to have increased contemporary gene flow are found between adjacent populations. The two routes between non-adjacent populations to show higher levels of contemporary gene flow are from TX to SC (Δm = +0.0567) and from NC to NY (Δm = +0.0320).

**Table 3:** Temporal changes in gene flow (Δm) among all populations; values in italics denote gene flow routes under a linear stepping-stone; bolded values denote an increase in contemporary gene flow (+Δm) of >0.010. Populations on the left are sending gene flow into recipient populations listed above columns.

Changes in gene flow over time.							
	TX	FL	SC	NC	MD	NJ	NY
TX	-	*-0*.*0027*	**+0.0567**	-0.0051	-0.0081	-0.0021	-0.0323
FL	*-0*.*0209*	-	*+0*.*0009*	-0.0118	-0.0051	-0.0060	-0.0067
SC	-0.0382	*-0*.*0026*	-	*-0*.*0441*	-0.0233	-0.0217	-0.0678
NC	-0.0215	-0.0074	***+0*.*1282***	-	***+0*.*0290***	-0.0193	**+0.0320**
MD	-0.0911	-0.0197	-0.0201	*-0*.*0148*	-	-0.0306	-0.0453
NJ	-0.0498	-0.0093	-0.0032	-0.0621	***+0*.*0137***	-	***+0*.*0738***
NY	-0.0194	-0.0172	-0.0011	-0.0217	+0.0006	*+0*.*0089*	-

### Models of population structure

As the diamondback terrapin has a linear distribution (Fig 1), we used a stepping stone process as our null model of population connectivity (Fig 2A). To model translocation events, we modified the stepping stone model to include unidirectional gene flow between TX and SC, and NC and NY; these models differed only in their relative isolation of FL (Fig 2B–2F). We modeled the Suwannee Seaway by modeling bidirectional gene flow between TX and SC, with FL either completely isolated (Fig 2G) or a sink population (Fig 2H). We found that the Atlantic Exchange model (Fig 2E), which modeled translocation events from TX to SC and NC to NY (gene flow between non-adjacent populations), and which included bidirectional gene flow between SC and FL but unidirectional gene flow between TX and FL, vastly outperformed all other models, including the linear stepping-stone model (Bézier scores = -13,7232 vs. -72,456.30). Converting approximate Bézier scores into posterior probabilities confirmed the Atlantic Exchange model as the best-fit model (PP = 100%). All other models had estimated posterior probabilities near 0%.

### Bottlenecks

We recovered no evidence for genetic bottlenecks for any sampling locality with the TPM or SMM, although the TPM for SC6 approached significance (Table 4; P = 0.055). Mode-shift tests also failed to detect bottlenecks for any sampling locality. In addition, neither the TPM, SMM, nor a mode-shift test detected bottlenecks for any STRUCTURE cluster (Table 4).

**Table 4:** BOTTLENECK results by sampling locality and STRUCTURE cluster. Shown are *P*-values for a genetic bottleneck under the SMM and TPM. An “L-Shaped” distribution under a Mode-Shift test indicates a bottleneck was not detected.

Bottleneck tests along the seaboards.			
	SMM	TPM	Mode-Shift
Sampling Locality			
SC1	0.578	0.344	L-Shaped
SC2	0.281	0.922	L-Shaped
SC3	0.422	0.945	L-Shaped
SC4	0.500	0.281	L-Shaped
SC5	0.656	0.500	L-Shaped
SC6	0.281	0.055	L-Shaped
NC	0.422	0.219	L-Shaped
MD	1.000	1.000	L-Shaped
NJ	0.922	0.719	L-Shaped
NY	0.945	0.922	L-Shaped
TX	0.719	0.781	L-Shaped
FL	0.961	0.961	L-Shaped
STRUCTURE Cluster			
SC	0.961	0.422	L-Shaped
MD	1.000	1.000	L-Shaped
NJ/NY/NC	0.961	0.719	L-Shaped
TX	0.719	0.781	L-Shaped
FL	0.961	0.961	L-Shaped

## Discussion

Over the last two centuries, the relationship between humans and terrapins has been complex, and the terrapin’s population genetic structure reflects this relationship. The demand for turtle soup resulted in historical population contractions and extirpations, and culminated in the construction of terrapin farms [3–6]. To get flavorful terrapins to market quickly, Texas and Carolina terrapins were hybridized at the North Carolina terrapin farm [6]. Then, in 1920, the enactment of Prohibition restricted access to sherry, which drastically cut demand for turtle soup. Consequently, many terrapins were released into local waters, which promoted population admixture and may have resulted in the reintroduction of genetic diversity.

We documented population genetic structure that is consistent with historical accounts of terrapin translocation during the twentieth century [1, 3]. We recovered two or three genotypic clusters in the mid- and north Atlantic (MD, NJ-NY) and three genotypic clusters in the Gulf and southern populations (TX, FL SC), for a total of six genotypic clusters (Fig 1B–1G; Fig 3). We also recovered the North American Gulf/Atlantic phylogeographic divide previously described in the terrapin and other taxa (Fig 1C) [8, 31, 35–37]. Recent work [38] has suggested that sampling localities with uneven sampling may lead to erroneous STRUCTURE results. While this is a concern, we do not think it is affecting our data. The population of concern in our study (Texas) has been recovered in previous studies [9], and is supported by our DAPC results (Figs 6 and 7) and an additional PCA analysis (S2 Fig).

In addition to delineating population structure and quantifying population connectivity, we also found that a modified stepping-stone model with genetic exchange along the Atlantic seaboard and unidirectional gene flow from TX to SC and from NC to NY best describes terrapin population structure (Fig 2E). This model of population connectivity outperformed a linear stepping-stone model, as well as models of the Suwannee Seaway, a natural conduit of gene flow between Gulf and Atlantic populations (Fig 2). Furthermore, we found Florida populations to be divergent from neighboring populations (Fig 1B–1D and 1G; Figs 4 and 6; Table 3), which complements accounts that Florida terrapins were not translocated due to their inferior size and taste [3], as well as other studies that reported FL terrapins to be genetically distinct [9–11].

Our Bayesian model comparisons supported bidirectional gene flow between SC and FL, and unidirectional gene flow from TX to FL (the Atlantic Exchange model, Fig 2E), but did not support alternative models of connectivity, such as the Suwannee Seaway (Fig 2).

We found that NC is highly admixed with other mid- north Atlantic populations (Fig 1C–1F), which could be the result of human-mediated gene flow. For example, relative to historical levels of gene flow, we documented increased contemporary connectivity from NC to NY (Table 3), which is consistent with accounts of translocation [1]. North Carolina was the location of a terrapin breeding operation [4–6], while NY was the location of a large terrapin market [1]. With the volume of terrapins brought to market, it is possible some terrapins escaped or were released into local waters. North Carolina also exhibited increased contemporary gene flow into SC and Chesapeake Bay, MD (Table 3), consistent with a previous study that detected large increases of contemporary gene flow into Chesapeake Bay [12]. Finally, we observed admixture between TX and SC populations (Fig 1D and 1G; Fig 6), as well as increased levels of contemporary gene flow from TX into SC (Table 3), consistent with chronicled translocation and hybridization experiments [1, 6, 8].

Although we documented population genetic evidence of translocation between some non-adjacent populations, our study failed to find genetic evidence for some known instances of translocation. In particular, we did not detect increases in contemporary gene flow or admixture between TX and NC (Fig 1B–1D; Figs 4 and 6; Table 3). There are several possible explanations: translocation can fail [39, 40], and released terrapins from TX may not have successfully interbred with local populations in NC. Alternatively, our sampling in NC may have not included admixed localities (Fig 1A). We also did not find increased contemporary gene flow from MD to NY, although we detected admixture between these populations (Fig 1B–1F; Figs 4 and 6; Table 3). Terrapins from MD may not have been released into local waters in NY, or they may have failed to interbreed. Despite current weak demand for turtle soup, terrapins from the Chesapeake Bay region continue to be sold at markets in New York, often illegally [41, 42]. Although our Bayesian model tests cast doubt on the Suwannee Seaway (Fig 2) as a viaduct for genetic transmission in the southern portion of the terrapin’s range, it remains possible that long distance (coastal) dispersal and gene flow are structuring a portion of our populations. Despite a variety of potential biogeographic barriers, the Balkan Pond Turtle (*Mauremys rivulata*) exhibits a paucity of genetic variation across its range [43], suggesting it is capable of transoceanic gene flow. Infrequent but long-range coastal gene flow may also be occurring between Diamondback Terrapin populations.

It is well documented that terrapin populations historically underwent severe contractions [2], but we failed to detect any population bottlenecks in any region (Table 4). For example, the decline of terrapins from Chesapeake Bay is documented by shrinking harvests after decades of overexploitation [7], but we did not detect bottlenecks in this region. Indeed, previous work in Chesapeake Bay indicated terrapins exhibit relatively high levels of genetic diversity [9, 12]. It is possible that we failed to detect population bottlenecks because tests of heterozygosity excess have weak statistical power [44]. Alternatively, the failure to detect bottlenecks may be the consequence of terrapin translocation events reintroducing genetic diversity into populations. If true, the enactment of Prohibition may have inadvertently benefited the terrapin in two ways. The first was the collapse of the turtle soup market, which slowed the harvesting of natural populations. The second was the closure of terrapin farms and the release of translocated individuals into local populations, which may have reintroduced genetic diversity and increased population viability [45].

Thus, our study suggests that population genetic structure in the diamondback terrapin possesses the signature of historical translocation events. Translocation among natural populations is known to increase levels of population admixture and genetic diversity [46, 47]. At least in some cases, increased levels of genetic diversity save populations from the negative consequences of inbreeding depression and lowered mean population fitness [48–52]. However, translocated populations may exhibit high levels of genetic diversity but have low effective population sizes, suggesting several population genetic metrics are required to judge the efficacy of translocation [53]. An alternative outcome of translocation is that it may harm populations by causing outbreeding depression, harm locally adapted populations by moving them away from an adaptive peak [54, 55], or introduce diseases [56]. Nonetheless, given the extent of the current biodiversity crisis [57–60], including increasing population fragmentation [61–64] conservation-oriented translocation has become increasingly pivotal to maintaining population viability [65].

Historically, terrapins have been divided into seven subspecies [8], but recent genetic studies are incongruent with extant taxonomy [9–11]. While our analyses also cast doubt on current terrapin taxonomy, we do not make recommendations for future taxonomic changes. Our study lacked sampling in the Gulf of Mexico, where there may be more population structure than currently documented. Our study also used six microsatellite loci; more thorough geographical sampling and additional loci are required to make recommendations on taxonomic changes.

Our study shows that convoluted genetic histories can be disentangled with modern population genetic tools and that translocation can leave an indelible fingerprint in populations. Anthropogenic influences increasingly disrupt population dynamics [12, 61, 62, 66]; however, the indirect consequences of social and political activities are not always predictable. Our study suggests the population genetic structure in the Diamondback Terrapin may be the byproduct of an interaction between market demand for turtle soup during the late nineteenth and early twentieth centuries, followed by the enactment of Prohibition in 1920, which resulted in the large scale release of captive terrapins into local waters.

## Supporting information

S1 Fig Likelihood scores for north Atlantic populations. Overlapping likelihood scores for the number of population in the north Atlantic (Fig 1E and 1F).
(TIFF)

S2 Fig PCA analysis of terrapin genotypes. PCA analysis of sampling localities. Location number corresponds with Figs 4 and 6.
(EPS)

S1 Text Raw data. Dataset with all terrapin genotypes.
(XLSX)

S2 Text Population structure files. STRUCTURE/adegent input files.
(STRU)

S3 Text Contemporary gene flow. BAYESASS input file.
(TXT)

S4 Text Historical gene flow. MIGRATE input file.
(TXT)

S5 Text Bottleneck file. BOTTLENECK input file, sampling localities.
(TXT)
