# Supplementary figures and images for: Turtle soup, Prohibition, and the population genetic structure of Diamondback Terrapins (Malaclemys terrapin)
Source: PLoS One. 2017 Aug 9;12(8):e0181898. doi: 10.1371/journal.pone.0181898 (PMC5549917; doi:10.1371/journal.pone.0181898)

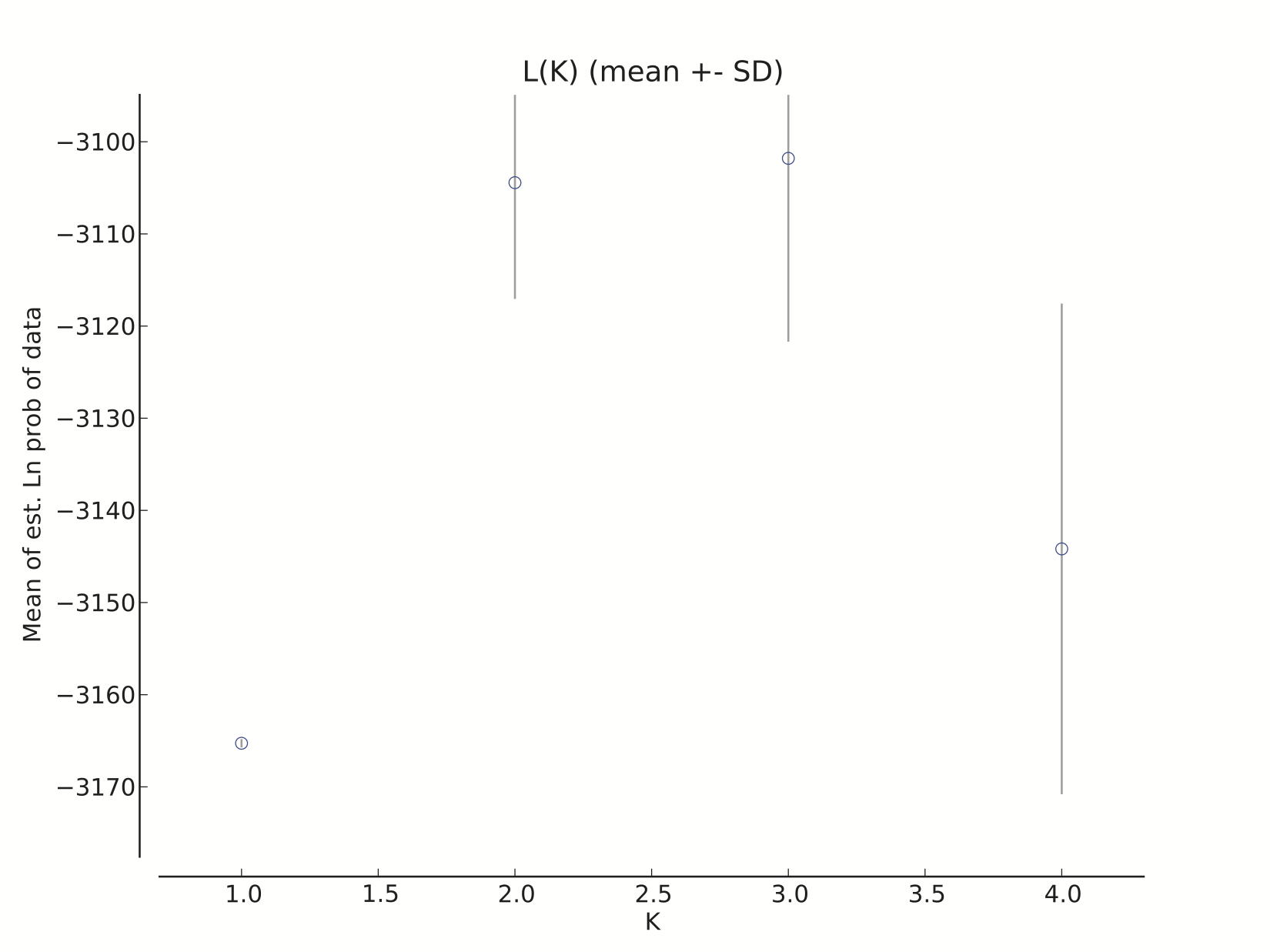

Supplement: S1 Fig — Overlapping likelihood scores for the number of population in the north Atlantic (Fig 1E and 1F). (TIFF) [file pone.0181898.s001.tiff]
